# Editorial: New Approaches to Investigate Congenital Vestibular Disorders

**DOI:** 10.3389/fneur.2022.921007

**Published:** 2022-05-30

**Authors:** François Simon, Kenna Peusner, Richard Lewis, Mathieu Beraneck

**Affiliations:** ^1^Université Paris Cité, Integrative Neuroscience & Cognition Center, CNRS UMR 8002, Paris, France; ^2^Department of Paediatric Otolaryngology, AP-HP, Hôpital Necker-Enfants Malades, Paris, France; ^3^Department of Neurology, The George Washington University School of Medicine and Health Sciences, Washington, DC, United States; ^4^Department of Otolaryngology-Head and Neck Surgery, Massachusetts Eye and Ear, Boston, MA, United States; ^5^Department of Otolaryngology-Head and Neck Surgery, Harvard Medical School, Boston, MA, United States

**Keywords:** childhood balance disorders, vestibular prostheses, gene therapy, animal models, congenital—diagnosis, etiology, balance, vestibular

For some, the vestibular system is so poorly understood that it is thought of simply as an accessory of the auditory system. As we have gained better understanding of this sensory system, we have learned that the vestibular system goes far beyond maintenance of posture and balance, participating in orientation in space, navigation, motor coordination, and body perception.

The mature inner ear consists of a labyrinth of diverse sensory organs set within a finely organized spatial configuration. Vestibular sensory organs detecting angular acceleration reside in the ampullae of three orthogonal semicircular canals, while changes in gravity and linear acceleration are detected primarily by the maculae utriculi and sacculi in the vestibule. All vertebrates maintain body equilibrium in space for quality of life and survival. These basic requirements may explain why the phylogenetically old vestibular system is highly conserved, with all vertebrates sharing a common blueprint for basic inner ear configuration and central vestibular pathways. An accurate control of posture, balance, gaze, and their perception requires integrating diverse sensorimotor signals within a single central nervous system framework. Spatially and temporally precise signals from the vestibular sensory organs are transmitted over widespread areas starting mainly with the brainstem vestibular nuclei where the first level of sensory integration occurs. Signaling then proceeds to other brainstem centers: the thalamus, cerebral cortex, cerebellum, and spinal cord.

Despite the evolutionary importance of these labyrinths for survival, malformation is not rare during development. Epidemiological studies report that up to 8% of children experience vertigo or balance problems (1), with vestibular disorders affecting about 3.3 million children in the US alone (2). The prevalence of pediatric vestibular dysfunction is probably underestimated because of difficulties in testing children, their rapid capacity for central multisensory compensation, and limited access to the pediatric population for vestibular investigations.

During the intense developmental period of childhood, vestibular disorders may produce similar deficits observed in adults, lesser problems due to more effective vestibular compensation in children, or more severe problems because some vestibular functions emerge before birth or during the 1st months of life, such as multisensory integration, basic postural reflexes, motor coordination, and gaze stability. The most obvious consequences of CVDs are hypotonia and delayed verticalization impacting sitting, standing, and the acquisition of walking. CVD children may naturally implement compensatory behaviors using visual or somesthetic cues, such as moving on the back to increase surface contact. Visual stability may be disturbed in CVD patients who experience decreased dynamic visual acuity and oscillopsia resulting in reading difficulties. These deficits induce fatigue in children and reduce their ability to concentrate that altogether hinder learning. Finally, vestibular deficits are associated with increased incidence of psychiatric comorbidities in CVD children (3).

The objective of this Research Topic is to describe clinical testing approaches and the results obtained in CVD children and CVD animal models presently available to better understand the pathology within central and peripheral vestibular system structures that produce behavioral deficits in CVD children. The study “*Peripheral Vestibular Dysfunction Is a Common Occurrence in Children With Non-syndromic and Syndromic Genetic Hearing Loss*” by Wang et al. reports new data indicating that vestibular disorders are commonly found in children with syndromic and non-syndromic hearing loss. Two other studies focus on new approaches for clinical exploration in CVDs. An enlarged vestibular aqueduct is one of the first malformations in CVDs, an anomaly that may be explored using the video head impulse test (VHIT), a caloric test described in “*Clinical Implication of Caloric and Video Head Impulse Tests for Patients With Enlarged Vestibular Aqueduct Presenting With Vertigo*” by Li et al. Imaging is a recent major development in vestibular testing, especially for Meniere's disease. This topic is discussed in “*Three-Dimensional Volumetric Measurement of Endolymphatic Hydrops in Meniere's Disease*” by Noh et al., paving the way for new discoveries in CVDs where endolymphatic MRI may contribute to understanding the underlying mechanisms in children.

Concerning animal models, “*Sustained Loss of Bdnf Affects Peripheral but Not Central Vestibular Targets*” by Elliott et al. describes the effect of brain-derived neurotrophic factor (BDNF) on vestibular ganglion neurons and vestibular hair cells in a mouse model. A translational study “*Vestibular Deficits in Deafness: Clinical Presentation, Animal Modeling, and Treatment Solutions*” by Maudoux et al. describes non-syndromic, vestibular impairments and how animal models are leading to promising results in treatments. Lastly, the paper “*Understanding the Pathophysiology of Congenital Vestibular Disorders: Current Challenges and Future Directions*” by Peusner et al. reports on the challenges faced by children with syndromic CVDs and how animal models can be used to acquire more precise assessment of the vestibular pathophysiology underlying the disorders at the cellular level.

## Author Contributions

All authors listed have made a substantial, direct, and intellectual contribution to the work and approved it for publication.

## Conflict of Interest

The authors declare that the research was conducted in the absence of any commercial or financial relationships that could be construed as a potential conflict of interest.

## Publisher's Note

All claims expressed in this article are solely those of the authors and do not necessarily represent those of their affiliated organizations, or those of the publisher, the editors and the reviewers. Any product that may be evaluated in this article, or claim that may be made by its manufacturer, is not guaranteed or endorsed by the publisher.

## References

[1] Niemensivu R Pyykkö I Wiener-Vacher SR Kentala E. Vertigo and balance problems in children–an epidemiologic study in Finland. Int J Pediatr Otorhinolaryngol. (2006) 70:259–65. 10.1016/j.ijporl.2005.06.015 16102845

[2] Li C-M Hoffman HJ Ward BK Cohen HS Rine RM. Epidemiology of dizziness and balance problems in children in the United States: a population-based study. J Pediatr. (2016) 171:240–7.e1–3. 10.1016/j.jpeds.2015.12.002 26826885

[3] Bigelow RT Semenov YR Hoffman HJ Agrawal Y. Association between vertigo, cognitive and psychiatric conditions in US children: 2012 National Health Interview Survey. Int J Pediatri Otorhinolaryngol. (2019) 2019:109802. 10.1016/j.ijporl.2019.109802 31809971 PMC7008084

